# Prognostic significance of the extent of septal fibrosis quantified on late gadolium enhanced images in patients with nonischemic cardiomyopathy

**DOI:** 10.1186/1532-429X-17-S1-O35

**Published:** 2015-02-03

**Authors:** Aidan K Cornhill, Yoko Mikami, Vijay Kandalam, Sebastien X Joncas, Irene Pauchard, Michael Bristow, Naeem Merchant, Tracy L Elliot, Carmen P Lydell, Andrew G Howarth, James A White

**Affiliations:** 1 Stephenson Cardiac Imaging Centre, Libin Cardiovascular Institute of Alberta, University of Calgary, Calgary, AB, Canada; 2 Diagnostic Imaging, University of Calgary, Calgary, AB, Canada; 3 Cardiac Sciences, University of Calgary, Calgary, AB, Canada; 4 Radiologie am Graben, Winterthur, Switzerland

## Background

Midwall septal fibrosis on late gadolinium enhancement (LGE) cardiovascular magnetic resonance (CMR) imaging has been shown to be an independent predictor of adverse events in patients with non-ischemic dilated cardiomyopathy (NICM). Recent studies in other cardiomyopathy cohorts, such as Hypertrophic Cardiomyopathy, suggest that LGE extent provides incremental prognostic value over its binary presence or absence. The objective of this study was to investigate the prognostic value of septal LGE quantification for the prediction of arrhythmic events among patients with NICM.

## Methods

A total of 122 consecutive patients with NICM (72 male, mean age 57±14yrs), defined as a LVEF<55% and no history of myocardial infarction, revascularization, or ischemic LGE by MRI were followed for the occurrence of sudden cardiac death (SCD) or appropriate implantable cardiac defibrillator (ICD) therapy. CMR imaging was performed using a 3T MRI scanner using a standard imaging protocol. All image analysis was blindly performed using commercially available software (CVI^42^, CircleCVI). Left ventricular volumes and function were determined from SSFP cine images. The binary presence or absence of mid-wall septal LGE was determined by an expert reader. A separate blinded observer quantified total Septal LGE mass using a ≥5SD signal threshold. Strict anatomic criteria were used to define the septal region for all analyses.

## Results

The mean LV EF was 32 ± 12%. At a median follow-up of 675 days 16 patients had suffered the primary outcome. The visual presence of Septal LGE was scored in 50% among those with an event versus 21% among those without event (HR 4.7). Septal LGE mass was significantly higher among those having an event versus those without (4.53±4.54g vs 1.73±2.48g, p=0.014, HR 1.2 per 1g). Multivariate analysis inclusive of the 2 variables of Septal LGE mass and LVEF showed Septal LGE mass to be an independent predictor of the primary outcome (HR 1.23 per 1g, p<0.001). ROC analysis of Septal LGE mass was performed (AUC 0.74) with a threshold of 2.67g providing optimal sensitivity (0.69) and specificity (0.83). Using this threshold those with high Septal LGE mass (N=29) were at a markedly increased risk of sudden cardiac death or appropriate ICD therapy (HR=16.01).

## Conclusions

Signal threshold based quantification of Septal LGE in NICM patients provides superior prediction of sudden cardiac death or appropriate ICD therapy versus its binary scoring (HR 16.0 versus 4.7). LGE quantification may be a valuable tool for the risk stratification of patients with NICM.

## Funding

This study was funded in part by: Heart and Stroke Foundation of Canada (NA 6488 Ontario research Fund - Imaging in Cardiovascular Therapeutics), Canada Foundation for Innovation, and Calgary Health Trust.

**Figure 1:**
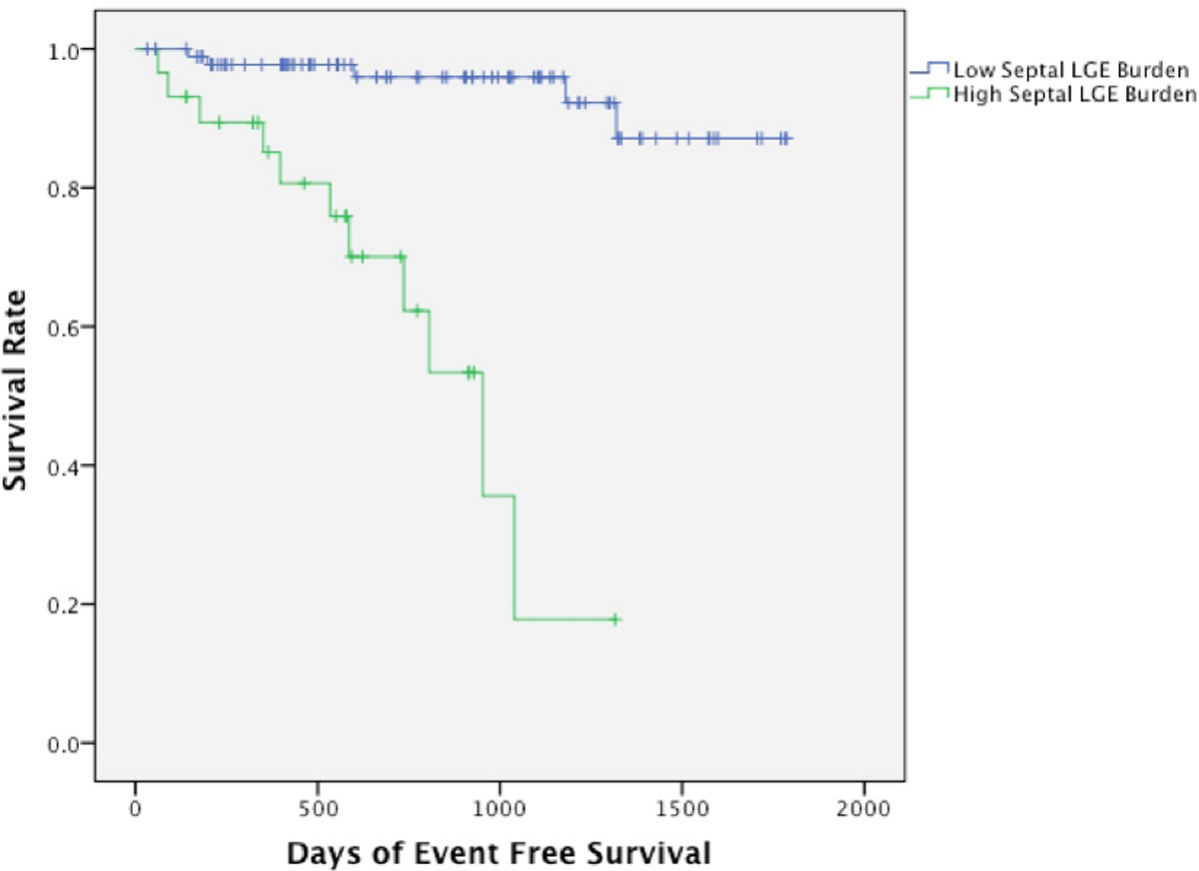
Kaplan-Meier survival curve showing the difference in survival rate between patients stratified into high Septal LGE mass (>2.67g) and low Septal LGE mass. Patients with high Septal LGE mass were at a markedly increased risk of primary outcomes.

